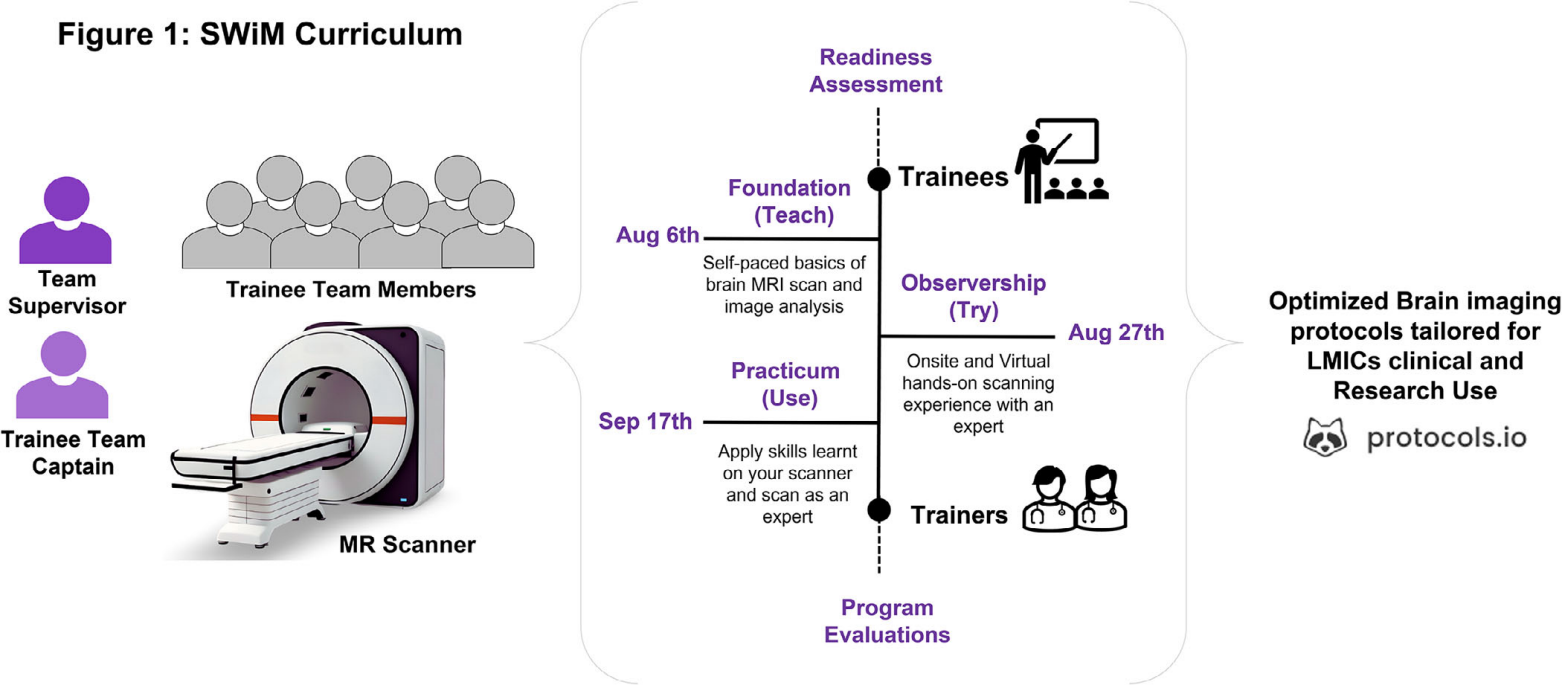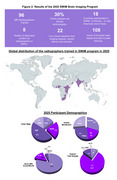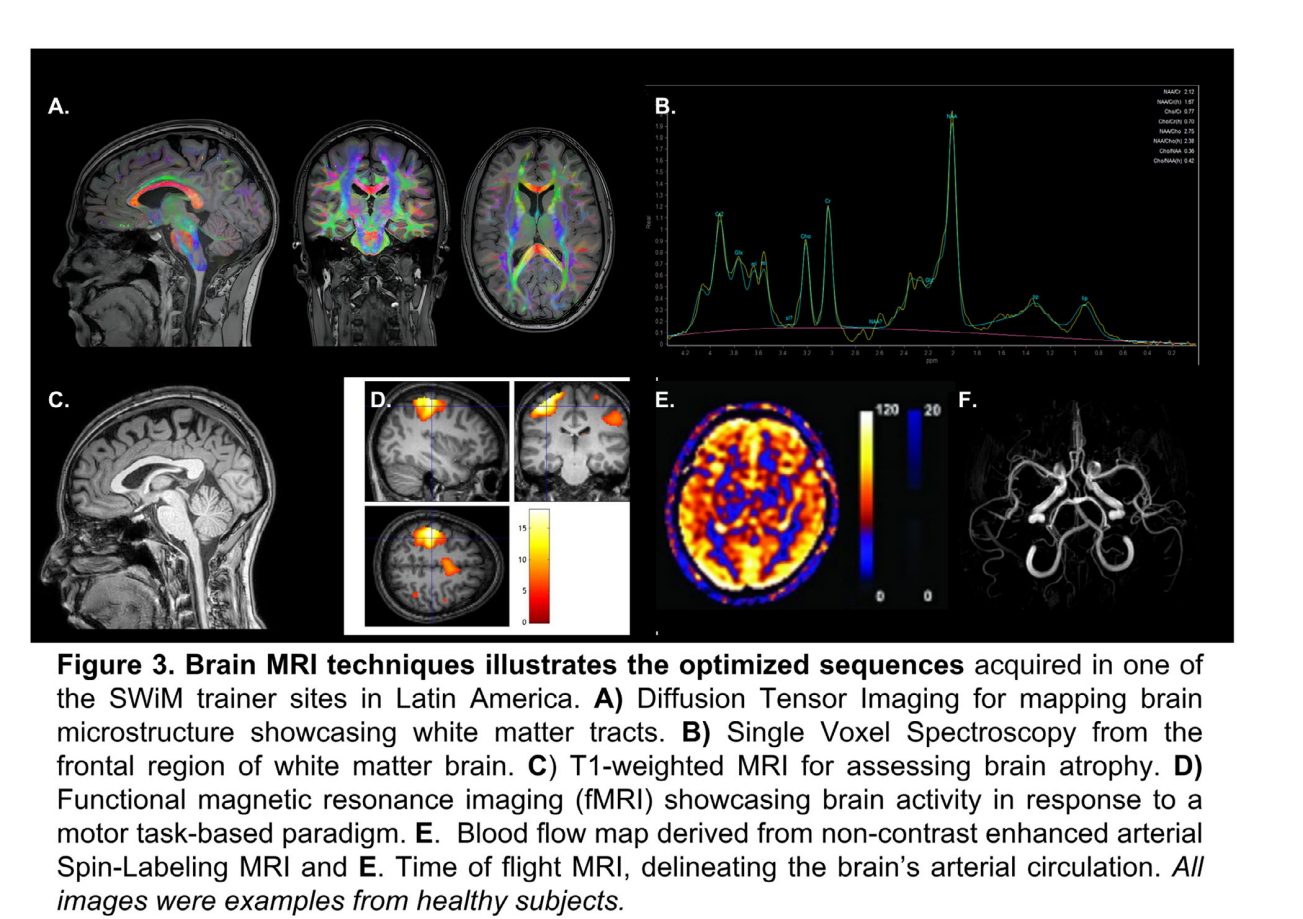# Enhancing Dementia Imaging in Low‐and‐Middle Income Countries Through Training of Skilled MRI Personnel

**DOI:** 10.1002/alz70862_109998

**Published:** 2025-12-23

**Authors:** Cristian Montalba, Harrison Aduluwa, Marina Fernandez Gracia, Francis Botwe, Surendra Maharjan, Abdul Nashirudeen Mumuni, Abderrazek Zeraii, Jackline Thairu, Sekinat Zurakat‐Aderibigbe, Adewale Adewo, Karabo Mokoena, Alexander Alfaro, Gonzalo Flores, Tchoyoson Lim, Mathieu Mach, Azeez Adebimpe, Reggie Taylor, Chinedum Anosike, Benjamin Anyanwu, Guy Poloni, Fatade Abiodun, Farouk Dako, Udunna Anazodo

**Affiliations:** ^1^ Biomedical Imaging Center, Pontificia Universidad Católica de Chile, Santiago, Santiago Chile; ^2^ Montreal Neurological Institute, McGill University, Montreal, QC Canada; ^3^ Valencia Polytechnic University, Valencia Spain; ^4^ University of Sussex, Brighton UK; ^5^ Department of Radiology, Weill Cornell Medicine, New York, NY USA; ^6^ University for Development Studies, Tamale Ghana; ^7^ Biophysics department, Higher Institute of Medical Technologies of Tunis, Tunis Tunisia; ^8^ Sonar Imaging Centre, Nairobi Kenya; ^9^ Department of Radiography, College of Medicine, University of Lagos, Lagos Nigeria; ^10^ Diamed Centre, Lagos Nigeria; ^11^ Steve Biko Academic Hospital, Pretoria South Africa; ^12^ Hospital Clínica Bíblica, San Jose Costa Rica; ^13^ Hospital Nacional Rosales, San Salvador El Salvador; ^14^ National Neuroscience Institute, Tan Tock Seng Singapore; ^15^ C.J.Gorter MRI Center, Leiden University Medical Center, Leiden Netherlands; ^16^ Bayer Corporation, Philadelphia, PA USA; ^17^ Brain Imaging Centre, Royal Mental Health Centre, Ottawa, ON Canada; ^18^ Accuread Radiology, Nigeria Ltd, Lagos Nigeria; ^19^ Regions Healthcare, Owerri Nigeria; ^20^ Siemens Healtineers, Erlangen Germany; ^21^ Crestview Radiology Ltd, Lagos Nigeria; ^22^ Perelman School of Medicine, University of Pennsylvania, Philadelphia, PA USA; ^23^ Medical Artificial Intelligence (MAI) Laboratory, Crestview Radiology Limited, Lagos Nigeria

## Abstract

**Background:**

In tandem with the ever‐increasing aging population in low‐ and middle‐ income countries (LMICs), the burden of dementia is rising across LMICs. Magnetic resonance imaging (MRI) is essential in diagnosis to evaluate different dementia subtypes. However, most LMICs have limited access to MRI and lack trained MRI personnel to make accurate diagnoses. Here, we provide update on the Scan With Me (SWiM) training program [1] aimed at upskilling MRI radiographers from LMICs to optimize MRI acquisition on their limited infrastructure and produce high‐quality images including advanced dementia MRI techniques.

**Method:**

SWiM is a free train‐the‐trainer capacity‐building initiative of the Consortium for Advancement of MRI Education and Research in Africa (CAMERA). SWiM implements RAD‐AID’s Teach‐Try‐Use strategy, which combines virtual learning resources, live expert case‐based lectures, and hands‐on vendor‐led practical scanning sessions to train a team of radiographers who work together as a network to enhance their skills and collectively train others [2]. The curriculum (Figure 1) guides participants from basic to advanced brain imaging over 8 weeks. Two cases of patients with dementia, simulated from LMIC published case reports were used for as capstone projects. This guided participants to develop brain imaging protocols tailored from standards (e.g., ADNI) and optimized on their scanners for dementia imaging.

**Result:**

The second program ran from August to September 2024, with special focus on dementia imaging and hands‐on imaging sessions at 5 clinics in Kenya and Nigeria, and one trainer site in Chile. 96 radiographers from 29 imaging facilities in 18 LMIC countries received 70 hours of specialized training, observerships, and peer‐to‐peer engagements (Figure 2). Basic to advanced MRI techniques adapted from ADNI were acquired from the two African sites using optimized protocols (Figure 3). The LMIC‐optimized scan protocols from participants are being curated and will be shared openly on protocols.io for others to use.

**Conclusion:**

SWiM aims to establish a collaborative network of experts in imaging centers in LMICs, enabling them to collect robust datasets that can inform clinical care and support the development of imaging tools for advancing prevention and treatment strategies.

[1] event.fourwaves.com/swim

[2] Mumuni AN, et al.,. J Am Coll Radiol. 2024 21(8):1222‐1234